# Effects of full replacement of dietary fishmeal with insect meal from *Tenebrio molitor* on rainbow trout gut and skin microbiota

**DOI:** 10.1186/s40104-021-00551-9

**Published:** 2021-02-03

**Authors:** Genciana Terova, Elisabetta Gini, Laura Gasco, Federico Moroni, Micaela Antonini, Simona Rimoldi

**Affiliations:** 1 Department of Biotechnology and Life Sciences, University of Insubria, Via J.H. Dunant, 3, 21100 Varese, Italy; 2 Department of Agricultural, Forest and Food Sciences, University of Turin, Largo P. Braccini 2- 10095 Grugliasco, Torino, Italy

**Keywords:** Aquaculture, Circular economy, Gut microbiome, Insect meal, Metagenome, Next-generation sequencing, Rainbow trout, Skin microbiome, *Tenebrio molitor*

## Abstract

**Background:**

Aquaculture must continue to reduce dependence on fishmeal (FM) and fishoil in feeds to ensure sustainable sector growth. Therefore, the use of novel aquaculture feed ingredients is growing. In this regard, insects can represent a new world of sustainable and protein-rich ingredients for farmed fish feeds. Accordingly, we investigated the effects of full replacement of FM with *Tenebrio molitor* (TM) larvae meal in the diet of rainbow trout (*Oncorhynchus mykiss*) on fish gut and skin microbiota.

**Methods:**

A feeding trial was conducted with 126 trout of about 80 g mean initial weight that were fed for 22 weeks with two isonitrogenous, isolipidic, and isoenergetic extruded experimental diets. Partially defatted TM meal was included in one of the diets to replace 100% (TM 100) of FM, whereas the other diet (TM 0) was without TM. To analyse the microbial communities, the Illumina MiSeq platform for sequencing of 16S rRNA gene and Qiime pipeline were used to identify bacteria in the gut and skin mucosa, and in the diets.

**Results:**

The data showed no major effects of full FM substitution with TM meal on bacterial species richness and diversity in both, gut mucosa- and skin mucus-associated microbiome. Skin microbiome was dominated by phylum Proteobacteria and especially by Gammaproteobacteria class that constituted approximately half of the bacterial taxa found. The two dietary fish groups did not display distinctive features, except for a decrease in the relative abundance of *Deefgea* genus (family Neisseriaceae) in trout fed with insect meal. The metagenomic analysis of the gut mucosa indicated that Tenericutes was the most abundant phylum, regardless of the diet. Specifically, within this phylum, the Mollicutes, mainly represented by Mycoplasmataceae family, were the dominant class. However, we observed only a weak dietary modulation of intestinal bacterial communities. The only changes due to full FM replacement with TM meal were a decreased number of Proteobacteria and a reduced number of taxa assigned to Ruminococcaceae and Neisseriaceae families.

**Conclusions:**

The data demonstrated that TM larvae meal is a valid alternative animal protein to replace FM in the aquafeeds. Only slight gut and skin microbiota changes occurred in rainbow trout after total FM replacement with insect meal. The mapping of the trout skin microbiota represents a novel contribution of the present study. Indeed, in contrast to the increasing knowledge on gut microbiota, the skin microbiota of major farmed fish species remains largely unmapped but it deserves thorough consideration.

## Introduction

Aquafeeds have largely been relied on fishmeal (FM), which is an optimal protein source to ensure fast growth and good health of farmed fish. However, most wild capture fisheries are operating at or above maximum sustainable yield; therefore, fish farming can no longer rely on oceanic resources for manufacturing aquafeeds and such feed options are simply not sustainable. This has promoted the search for more sustainable alternative ingredients to reduce the inclusion of FM in aquafeeds.

In this regard, insects can represent a new world of sustainable and protein-rich ingredients for farmed fish feeds. Breeding insects has low environmental footprint and this makes them even more interesting as protein source for aquafeeds [1]. Furthermore, insects are very efficient and quick bio converters – which makes them excellent organic waste recyclers. They can grow on agricultural wastes [2, 3], such as expired fruit and vegetables from packaging facilities and convert them into their own biomass, i.e., a high-value protein resource for farmed animals (pig, chicken, and fish) [1]. There is a real potential here to convert millions of tons of agricultural waste produced globally each year, into tones of high quality proteins for fish feeds [4], which in turn can increase fish production for human consumption, thus improving food and nutrition security, promoting economic growth and protecting our environment and natural resources

Demonstrating the emergence of a new sector, in recent years, a bulk of research has focused on insects [5–9] and dozens of companies all over the Europe have started breeding insects.

In this view, the yellow mealworm, *Tenebrio molitor* (TM) (Coleptera: Tenebrionidae), is a great match because it is very efficient at bio converting organic waste - the ideal circular insect! Furthermore, the percentage of edible biomass in larval and pupal stages of TM is only slightly less than 100% [10]; therefore, low extra waste (insect excreta called frass), is produced following its rearing. Mealworm frass is considered a sustainable resource for managing plant nutrition in cropping systems and a promising alternative to conventional fertilizer [4, 11]. Frass can also be employed to grow earthworms such as *Lumbricus terrestris or Eisenia fetida*, which may improve the efficiency of organic fertilizers [4, 11].

*T. molitor* is one of the seven insect species (2 flies, 2 mealworms, and 3 cricket species) that has been recently authorized by an EU commission regulation (2017/893–24/05/2017) for fish feed. Larval and pupal stages of TM are rich in protein and lipids whose levels range from 47% to 60% and from 31% to 43% (on a dry weight basis), respectively. In terms of protein quality, meal from TM larvae has a well-balanced amino acid profile and the content of some indispensable amino acid is higher (as % of protein) than in land plants and slightly lower than in FM [12].

Different studies have successfully incorporated TM as a protein source in the diet of different fish species. In rainbow trout (*Oncorhynchus mykiss*), feeding trial using diets with different FM/TM meal replacement levels have shown optimal fish performance [13–15]. In red seabream (*Pagrus major*), significant growth enhancement was obtained in fish fed on diets with 65% defatted TM larvae meal, i.e., complete replacement of FM [16]. Furthermore, in a study conducted on Nile tilapia (*Oreochromis niloticus*), TM had the highest apparent digestibility coefficient in comparison to other four insect meals that were tested, validating TM larvae as a good protein source alternative to FM for fish diets [17].

Insects contain bioactive compounds that are able to modulate the vast consortiums of microorganisms that inhabit fish gut. Therefore, diets in which FM was replaced by insect meal from either *Hermetia illucens* or *T. molitor*, have led to changes in the diversity and abundance of fish gut bacteria [18–20]. Studies indicate that chitin, a major structural component of the insect cuticles, is a potential modulator of fish gut microbiota [21], as it acts as a substrate for chitinase producing bacteria that are not commonly found in the fish gut [22, 23]. Supplementation of chitin or krill (chitin-rich) in the diet of Atlantic salmon (*Salmo salar*) changed the membership and structure of intestinal microbiota with over a hundred autochthonous bacterial strains identified [24].

Much of the current research on fish microbiota has focused on the microbial communities present in the gut, but fish harbor distinct microbial communities across other major anatomical regions, too. Of these anatomical sites, the skin contains the highest microbial diversity, followed by gills and gut [25–30]. The skin of fish is covered with thin and partially overlapping scales for protection and secretes an aqueous mucus layer that coats the epidermal surface. All these structures and appendages, with an abundance of folds and invaginations provide many specialized skin niches that harbour a wide range of microorganisms [27]. Furthermore, skin mucus is a biochemically complex fluid that includes a number of nutrients that favour a high bacterial diversity.

In contrast to the increasing knowledge on gut microbiota, the skin microbiota of major farmed fish species remains largely unmapped but it deserves thorough consideration [31]. Indeed, skin is one of the main mucosal barriers between fish and its external environment, constituting the first line of defense from pathogens or toxic substances [27]. Fish inhabit an aqueous environment very rich in highly diverse planktonic microbes, including bacteria, fungi and viruses. Such microbial-rich surrounding environment has potential to colonize fish skin and cause infections [31]. Consequently, fish have evolved mechanisms to gain benefits from harmless symbiotic bacteria, which help them to fight against invasion by pathogenic or harmful microorganisms. For instance, fish skin mucus host commensal bacterial species, which are able to protect their host against pathogens by inhibiting enzymatic activities and secreting antimicrobial compounds [32]. Skin microbiota plays thus a critical role in the control of fish diseases. Therefore, an enhanced understanding of host-symbiont-pathogen nexus is necessary not only to gain insight into microbial involvement in fish diseases, but also to enable novel promicrobial and antimicrobial approaches for their treatment.

To the best of our knowledge, there are no articles in the literature dealing with the effects of diet on skin microbiota of farmed fish. However, since the feed catabolites are dispersed in the water, and the quality of water is one of the factors that can change the composition of fish microbiota [33–35], it would be interesting to see the dynamics of both gut and skin microbiota in fish fed diets with insect meal.

Accordingly, the present research aimed at investigating the effects of full replacement of FM with TM larvae meal in the diet of rainbow trout (*Oncorhynchus mykiss*) on fish growth performance, and microbiota of gut and skin. The feed microbiota was analyzed, too.

## Methods

### Feeding trial, diets and fish sampling

Details of the feeding trial have been described by Chemello et al. [36]. In brief, SPAROS LDA (Olhão, Portugal) and Ÿnsect (Evry, France) formulated two isonitrogenous, isolipidic, and isoenergetic extruded experimental diets named TM 0 and TM 100. Partially defatted TM meal was included in one of the diets to replace 100% (TM 100) of FM, whereas the other diet (TM 0) was without TM. Main ingredients and proximate composition of the diets are shown in Table 1. The processing and storage conditions of the two diets were the same. The feeds were stored in a refrigerated room (6 °C) for the entire duration of the feeding trial.

**Table 1:** Main ingredients and proximate composition of the diets

Item	TM 0	TM 100
Ingredients, %		
Fishmeal 65 (Peruvian)	20	-
*Tenebrio molitor* larvae meal	-	20
Soy protein concentrate	18	18
Wheat gluten	7.75	7.06
Corn gluten	8	8
Soybean meal (48%)	7	7
Wheat meal	14.23	13.8
Sardine oil	4.3	4.1
Soybean oil	8.6	8.2
Rapeseed oil	8.6	8.2
Soy lecithin	0.5	0.5
Vitamin^a^ and mineral premix^b^	1	1
Antioxidant	0.2	0.2
Sodium propionate	0.1	0.1
Monocalcium phosphate	0.52	1.73
*L*-Arginine	-	0.1
*L*-Lysine	-	0.6
*L*-Tryptophan	0.05	0.12
*DL*-Methionine	0.15	0.3
Celite®	1	1
Proximate composition, % as fed		
Dry matter	93.77	94.41
Crude protein	42.08	44.25
Ether extract	22.63	22.36
Ash	7.57	5.6
Chitin	-	1.49
Nitrogen-free extract^c^	21.49	20.71
Gross energy, MJ/kg as fed^d^	22.24	22.55

This table has been modified from previously published data in Chemello et al. [36]

^a^Vitamin mixture (IU or mg per kg diet): *DL*-αtocopherolacetate, 60 IU; sodium menadione bisulfate, 5 mg; retinylacetate, 15,000 IU; *DL*-cholecalciferol, 3000 IU; thiamin, 15 mg; riboflavin, 30 mg; pyridoxine, 15 mg; vitamin B_12_, 0.05 mg; nicotinic acid, 175 mg; folic acid, 500 mg; inositol, 1000 mg; biotin, 2.5 mg; calcium panthotenate, 50 mg; choline chloride, 2000 mg (Granda Zootecnici, Cuneo, Italy)

^b^Mineral mixture (g or mg per kg diet): bicalcium phosphate 500 g, calcium carbonate 215 g, sodium salt 40 g, potassium chloride 90 g, magnesium chloride 124 g, magnesium carbonate 124 g, iron sulfate 20 g, zinc sulfate 4 g, copper sulfate 3 g, potassium iodide 4 mg, cobalt sulfate 20 mg, manganese sulfate 3 g, sodium fluoride 1 g (Granda Zootecnici, Cuneo, Italy)

^c^Calculated as 100 − (crude protein + ether extract+ ash + chitin)

^d^Determined by calorimetric bomb

Rainbow trout of 78.3 ± 6.24 g mean initial weight were randomly distributed into six 400-L tanks (3 tanks/diet, 21 fish/tank). Tanks were supplied with artesian well water at 13 ± 1 °C in a flow-through open system (tank water inflow: 8 L/min). The dissolved oxygen levels were measured every 2 weeks and ranged between 7.6 and 8.7 mg/L, whereas the pH was 7.5–7.6. The feeding trial lasted 22 weeks. The first 8 weeks, fish were fed at 1.6% of the tank biomass and then, according to the fish growth and water temperature, the daily quantity of distributed feed was decreased to 1.4%. Fish were fed twice a day (at 8:00 and at 15:00), 6 d per week. Feed intake was monitored at each administration. In order to update the daily feeding rate, fish in the tanks were weighed in bulk every 14 days. Mortality was checked every day.

At the end of the trial, six fish/diet were sampled and the whole intestine was aseptically dissected out. The animals used for sampling were sacrificed by an overdose of anaesthetic (MS-222; PHARMAQ Ltd., UK; 500 mg/L) using water bath immersion and all efforts were made to minimize pain, stress, and discomfort in the animals. The skin mucus microbiota was obtained by gentle scraping of fish body with a cotton swab (individually wrapped sterile cotton swab with a polystyrene handle), whereas the gut autochthonous microbiota was obtained by scraping the mucosa of the entire intestine (excluding pyloric caeca). Each swab head was immediately cut off and placed inside a sterile 1.5 mL Eppendorf tube containing 200 μL of Xpedition Lysis/Stabilization Solution. The tube was then vortexed for shaking out the bacteria from the swab tip [18] and stored at room temperature for up to 24 h until bacterial DNA extraction. Trained researchers performed all collection procedures.

### Bacterial DNA extraction

The bacterial DNA was extracted from four aliquots from each feed, six samples of skin mucus, and six samples of intestinal mucosa per each dietary fish group. The DNA extraction from feeds was done in parallel to biological samples, right after the end of feeding trial.

DNeasyPowerSoil® Kit (Qiagen, Italy) was used to extract DNA, following the manufacturer’s instructions with only few modifications at the lysis step, as previously described by Rimoldi et al. [37]. In brief, 200 mg of feed or 200 μL of skin and gut bacteria suspension were lysed in PowerBead Tubes by means of a TissueLyser II (Qiagen, Italy) for 2 min at 25 Hz. A sample with only lysis buffer was processed in parallel to the biological samples as a negative control of the extraction procedure. The concentration of extracted DNA was measured using NanoDrop™ 2000 Spectrophotometer (Thermo Scientific, Italy). Then, bacterial DNA was stored at − 20 °C until the microbiota sequencing.

### Illumina 16S metagenomic sequencing library construction

16S ribosomal RNA gene amplicon libraries were prepared using a pair of primers specific for the V3-V4 region applying the Illumina protocol “16S Metagenomic Sequencing Library Preparation for IlluminaMiSeq System” (#15044223 rev. B). Amplicons of 16S rRNA gene were generated starting from 10 μL of microbial genomic DNA by PCR using Platinum®Taq DNA Polymerase High Fidelity (Thermo Fisher Scientific, Italy) and tailed forward and reverse primer Pro341F (5′-CCTACGGGNBGCASCAG-3′) and Pro805R (5′-GACTACNVGGGTATCTAATCC-3′) selected by [38] The expected size of PCR products on Agilent 2100 Bioanalyzer trace was ~550 bp. The entire procedure for 16S rRNA gene library preparation and sequencing is described in [18] In brief, Illumina paired-end adapters with unique Nextera XT indexes were ligated to 16S amplicons using Nextera XT Index Kit (Illumina, San Diego, CA, USA). A quality control of all libraries was then performed by qPCR using KAPA Library Quantification Kits Illumina® Platforms (KapaBiosystems Ltd, UK). Libraries were then pooled at equimolar concentrations and diluted to 6 pM. Pooled libraries were then multiplexed and sequenced on an Illumina MiSeq platform (Illumina) with paired-end 2 × 300 bp sequencing chemistry.

### Metagenome data analysis

Raw sequencing data were processed by QIIME 2 (2018.8) pipeline [39] at the default setting. Barcode sequences and primers were removed using the Cutadapt software v.2018.8.0 from raw reads. The sequences were filtered for quality (Q > 30), trimmed at the 3′ end and merged with default values of DADA2 software package. The remaining high quality reads were then dereplicated to obtain the unique sequences (*uniques*) and the chimeras were eliminated using qiime DADA2 denoise-paired command. The sequences were clustered in operational taxonomic units (OTUs) at 99% of similarity. The OTUs were filtered at 0.005% of frequency and two OTU-tables (one per each macro-group of samples: skin mucus+feeds and gut mucosa+feeds) were created. The rarefaction analysis was performed on the OTU-tables (biom format) to verify the minimum number of reads to normalize all samples. Each OTU was taxonomical assigned using GreenGenes v.13-8 as reference database. Reads assigned to chloroplasts and mitochondria were removed from the analysis since of eukaryotic origin. Alpha-diversity analysis was performed based on rarefied OTU tables considering Observed OTUs, Shannon, Pielou’s evenness, and Faith PD indices. To compute microbial beta diversity both weighted and unweighted UniFrac analyses were performed and sample UniFrac distances were visualized on 3D PCoA plots.

### Statistical analysis

The number of reads across samples was normalized by sample size and the relative abundance (%) of each taxon was calculated. Only those taxa with an overall abundance of more than 1% (up to order level) and 0.5% at family and genus level were considered for statistical analysis. Before being statistically analysed, the resulting microbial relative abundances were calculated as the angular transformation (arcsine of the square root). All data were checked for normality and homoscedasticity by Shapiro-Wilk’s and Levene’s test, respectively. Depending if normality of the data was satisfied or not, differences between groups were analysed by *t*-test or by nonparametric Mann-Whitney test. Statistical significance was set at *P<*0.05. All the statistical analyses were performed using Past4 software version 4.02 [40] . Kruskal-Wallis test was applied to verify differences in alpha-diversity indices between treatments. Multivariate analysis of beta diversity was verified using non parametric permutational multivariate analysis of variance (Adonis) and analysis of similarity (ANOSIM) with 999 permutations (*P<*0.05). Both alpha and beta metrics, including their related statistics, were computed using QIIME 2’s diversity analysis commands “qiime diversity alpha-group-significance” and “qiime diversity beta-group-significance” available through the q2-diversity plugin.

## Results

### Fish growth performance

Our previous publication by Chemello et al. [36] reported all data on fish growth performances and feed utilization efficiency. In brief, at the end of the feeding trial, all fish tripled their mean body weight, but there were no significant differences between the dietary groups for any of the considered growth performance indexes (*P* > 0.05). The mean individual weight gain was 312 g and 353 g for fish fed with TM 0 and TM 100 diets, respectively, whereas feed conversion ratio was 1.07 and 1.02, respectively. Protein efficiency rate was 2.09 for both dietary groups.

### Evaluation of microbiome diversity

Thirty-two microbiome profiles (from 8 feeds, 12 skin mucus, and 12 gut mucosa samples) were successfully obtained by high throughput sequencing of 16S rRNA gene amplicons on Illumina MiSeq platform. A total of 1,701,326 of reads were achieved, corresponding to 575 OTUs and 158 OTUs for skin mucus+feeds and gut mucosa+feeds macro-groups, respectively.

To calculate alpha diversity indices, samples were rarefied to 21,146 reads for gut mucosa+feeds macro-group and to 16,752 reads for skin mucus+feeds macro-group, but maintaining an adequate Good’s coverage (> 0.99). The number of OTUs ranged from 84 to 107 for feed-associate bacterial communities, from 9 to 13 for gut mucosa, and from 153 to 187 for skin mucus microbial community (Table 2). No statistically significant differences were found for any of the alpha diversity index considered, within the same starting sampling substrate, in response to diet (*P* ≥ 0.05). The only exception was represented by Shannon index value, which resulted significantly higher in TM 100 feed samples (*P* = 0.021). Although due to the different level of rarefaction, it is not statistically acceptable to compare the two anatomical districts (gut and skin) to each other, skin microbiome clearly showed higher bacterial species richness (Observed OTUs) and biodiversity (Shannon and Faith PD indices) than intestine. All sequencing data were deposited as FASTQ files at the European Nucleotide Archive (EBI ENA) public database under the accession code: PRJEB38845.

**Table 2:** Alpha diversity. Number of reads per group-treatment assigned to OTUs and alpha diversity metrics values of feed, gut mucosa (GMMC), and skin mucus microbial communities (SMMC) of rainbow trout fed TM 0 and TM 100 diets

Items	TM 0	TM 100	***P***-value
Feed (rarefied at 21,146 reads)			
Reads	54,465 ± 18,561	44,708 ± 19,771	0.498
Observed OTUs	107 ± 20	84 ± 25	0.248
Shannon	3.73 ± 0.05	3.29 ± 0.07	**0.021**
Pielou’s evenness	0.55 ± 0.02	0.52 ± 0.03	0.083
Faith PD	7.79 ± 0.68	6.70 ± 1.05	0.149
GMMC (rarefied at 21,146 reads)			
Reads	63,530 ± 31,477	61,665 ± 16,983	0.901
Observed OTUs	13 ± 3	10 ± 4	0.231
Shannon	1.32 ± 0.76	0.28 ± 0.24	0.054
Pielou’s evenness	0.36 ± 0.21	0.09 ± 0.07	0.055
Faith PD	1.34 ± 0.23	1.26 ± 0.42	0.872
SMMC (rarefied at 16,752 reads)			
Reads	49,824 ± 21,594	39,064 ± 16,875	0.359
Observed OTUs	187 ± 40	154 ± 71	0.336
Shannon	4.43 ± 1.05	4.29 ± 1.05	0.749
Pielou’s evenness	0.59 ± 0.13	0.61 ± 0.06	0.521
Faith PD	17.30 ± 4.52	13.86 ± 7.18	0.423

All data are expressed as means ± SD (*n* = 4 for feed and *n*=6 for GMMC and SMMC). *P<*0.05 are in bold

**Fig. 1:**
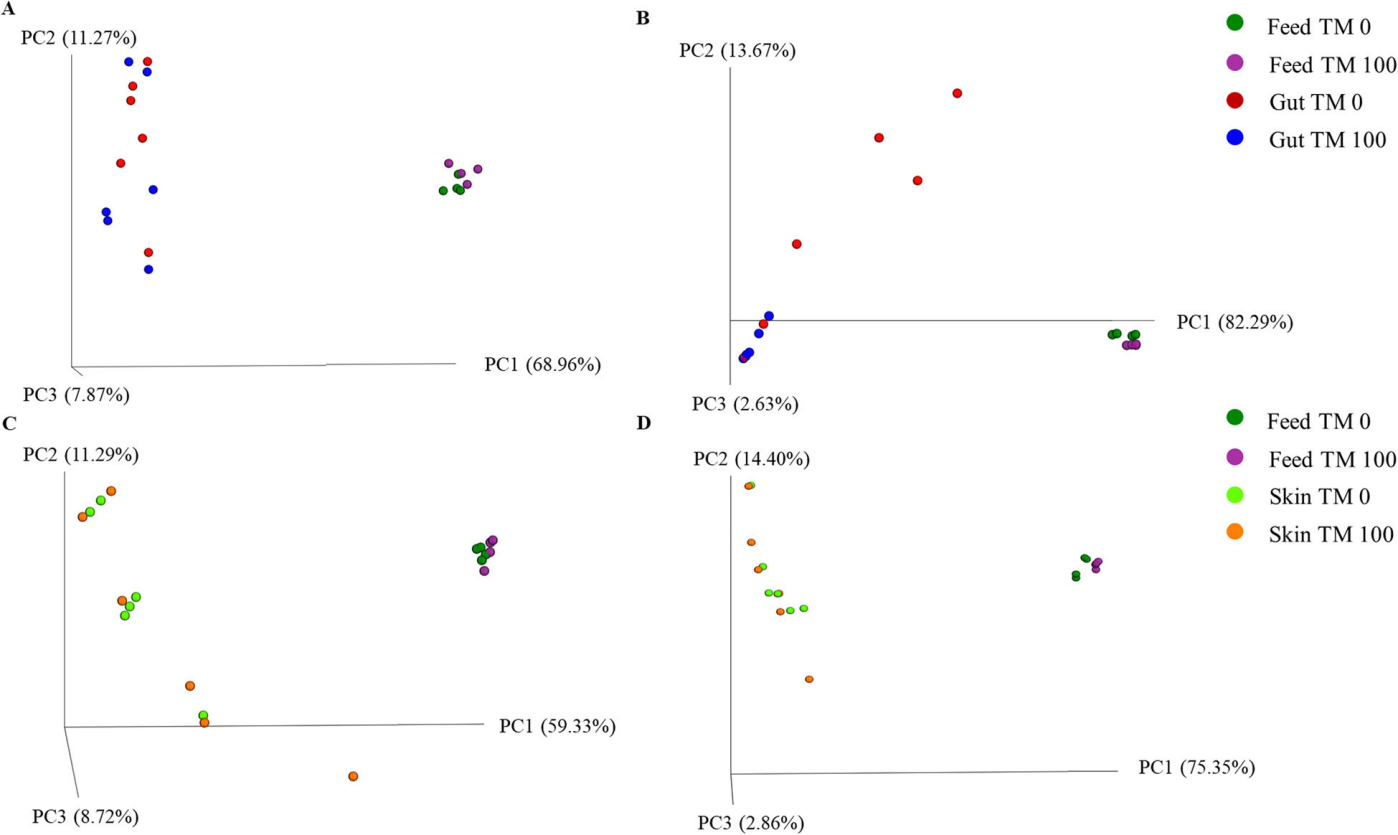
Unifrac PCoA plots. Unweighted, **a,** and Weighted, **b.** Unifrac PCoA of gut mucosa and feed-associate microbial communities; Unweighted, **c**, and Weighted, **d**, Unifrac PCoA of skin mucosa and feed-associate microbial communities. Each dot represents an individual sample according to its microbial profile

The multivariate analysis Adonis of feed microbial communities based on UniFrac distance matrix, showed differences between TM 0 and TM 100 diets in terms of presence/absence (unweighted UniFrac), and relative abundance (weighted UniFrac) of taxa (Adonis unweighted *P* = 0.038 and weighted *P* = 0.034) (Table 3). Significant differences were also found between microbial communities of gut mucosa in function of the diet, but in this case only for weighted UniFrac analysis (Adonis *P* = 0.025 and ANOSIM *P* = 0.038) (Table 3). On the contrary, the diet type seemed to exert no effect on microbial communities associate to skin mucus (Table 3). Accordingly, for both macro-groups of analysis, PCoA plots clearly showed that feed samples clustered separately from biological samples, thus indicating that observed differences were not simply a consequence of feed contamination that might have been present in the gastrointestinal tract or water (Fig. 1). Weighted Unifrac PCoA confirmed that the gut mucosa communities were the only affected by diet type (Fig. 1b).

**Table 3:** Beta diversity. Permutational multivariate analysis of variance (Adonis) and Analysis of similarity (ANOSIM) on weighted and unweighted UniFrac distances of feed, gut mucosa (GMMC), and skin mucus microbial communities (SMMC) at genus level

Items	Adonis				ANOSIM			
	Unweighted		Weighted		Unweighted		Weighted	
	***P***-value	***R***^**2**^	***P***-value	***R***^**2**^	***P***-value	***R***	***P***-value	***R***
Feeds								
diet TM 0 vs. diet TM 100	**0.038**	0.33	**0.034**	0.42	**0.023**	0.38	**0.029**	0.53
GMMC								
TM 0 vs. TM 100	0.315	0.11	**0.025**	0.37	0.339	0.04	**0.038**	0.25
TM 0 vs. diet TM 0	**0.004**	0.72	**0.007**	0.64	**0.005**	1.0	**0.008**	0.92
TM 100 vs. diet TM 100	**0.006**	0.66	**0.003**	0.99	**0.004**	1.0	**0.007**	1.0
SMMC								
TM 0 vs. TM 100	0.525	0.08	0.274	0.11	0.456	0.00	0.140	0.11
TM 0 vs. diet TM 0	**0.006**	0.73	**0.011**	0.86	**0.007**	1.0	**0.004**	1.0
TM 100 vs. diet TM 100	**0.004**	0.48	**0.003**	0.82	**0.011**	0.58	**0.005**	1.0

*P <* 0.05 are in bold

### Characterization of microbial community associated to feeds

**Fig. 2:**
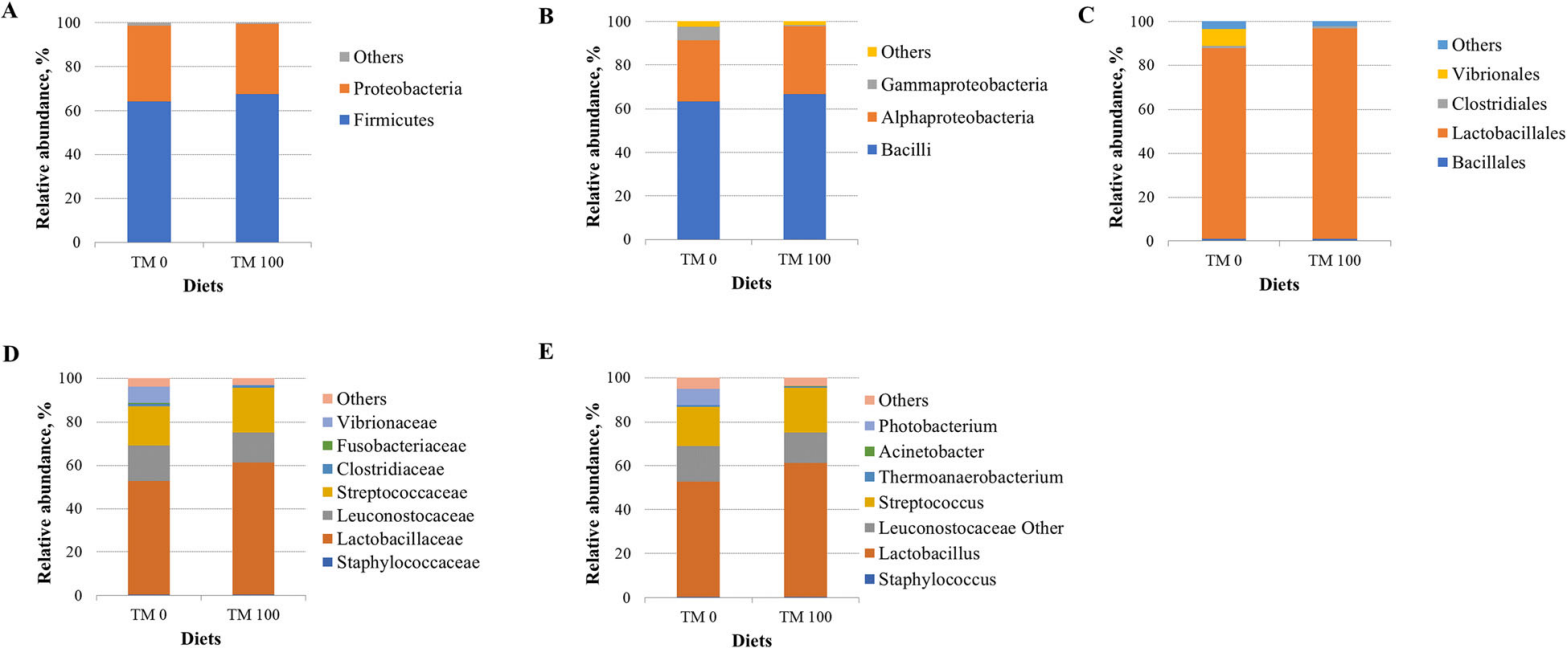
Relative abundance (%) of the overall most prevalent bacterial phyla (**a**), classes **(b**), orders (**c**), families (**d**), and genera (**e**) in feeds. All bacteria with an overall abundance of ≥1% for **a**, **b** and **c**; and ≥ 0.5% for **d** and **e** were reported. Bacteria with lower abundance were pooled and indicated as “Others”

Considering only the most representative taxa, the overall feed microbial community consisted of 2 phyla, 3 classes, 4 orders, 7 families, and 6 genera (Fig. 2; Table 4). At phylum level Firmicutes and Proteobacteria constituted together approximately 99% of bacteria population (Fig. 2a). Differences in taxa abundance were found at lower taxonomical levels. Feed TM 0 had more abundance of Gammaproteobacteria (3-fold increase, *P* = 0.030) compared to feed TM 100 containing insect meal (Fig. 2b, Table 4). At order level, Vibrionales were only found at consistent in percentage associate to diet TM 0 (*P* = 0.030), whereas, Lactobacillales were significantly (0.13-fold increase, *P* < 0.001) more abundant in feed TM 100 (Fig. 2c, Table 4). Accordingly, at family level, Vibrionacea*e* were practically undetectable in feed TM 100 (*P* = 0.030), resulting together with Fusobacteriaceae (6-fold increase, *P* = 0.026) and Staphylococcaceae (0.5-fold increase, *P* = 0.026) more abundant in control feed TM 0 (Fig. 2d; Table 4). Lactobacillaceae were enriched in feed TM 100 (0.21-fold increase, *P* = 0.006) (Fig. 2d; Table 4). The relative abundance of genus *Lactobacillus* was higher in TM 100 than in control feed (0.2-fold increase, *P* = 0.006), which was instead characterized by higher amount of *Photobacterium* (5-fold increase, *P* = 0.030) and *Staphylococcus* (0.5-fold increase, *P* = 0.038) genera (Fig. 2e; Table 4).

**Table 4:** Mean of relative abundance (%) ± SD of the most prevalent phyla, classes, orders, families, and genera found in feeds

Items	TM 0	TM 100	***P***-value
Phylum			
Firmicutes	64.20 ± 4.37	67.48 ± 3.14	0.271
Proteobacteria	34.54 ± 4.27	31.85 ± 3.15	0.351
Class			
Bacilli	63.39 ± 4.30	66.79 ± 3.24	0.254
Alphaproteobacteria	27.90 ± 3.66	30.96 ± 3.18	0.251
Gammaproteobacteria	6.30 ± 0.71	0.65 ± 0.12	**0.030**
Order			
Lactobacillales	86.77 ± 1.65	95.73 ± 0.28	**<0.001**
Vibrionales	7.50 ± 1.18	0.17 ± 0.29	**0.030**
Clostridiales	1.12 ± 0.20	0.99 ± 0.72	0.483
Bacillales	1.02 ± 0.01	0.98 ± 0.28	0.691
Family			
Lactobacillaceae	52.33 ± 2.97	60.95 ± 2.91	**0.006**
Streptococcaceae	17.95 ± 1.59	20.52 ± 4.35	0.470
Leuconostocaceae	16.25 ± 0.52	13.88 ± 1.88	0.055
Vibrionaceae	7.50 ± 1.18	0.17 ± 0.08	**0.030**
Clostridiaceae	0.99 ± 0.18	0.90 ± 0.26	0.553
Fusobacteriaceae	0.70 ± 0.21	0.01 ± 0.03	**0.026**
Staphylococcaceae	0.51 ± 0.06	0.39 ± 0.07	**0.038**
Genus			
*Lactobacillus*	52.22 ± 3.01	60.85 ± 2.89	**0.006**
*Streptococcus*	17.78 ± 1.60	20.24 ± 4.36	0.665
*Photobacterium*	7.44 ± 1.13	0.17 ± 0.08	**0.030**
*Thermoanaerobacterium*	0.70 ± 0.16	0.68 ± 0.19	0.885
*Staphylococcus*	0.51 ± 0.06	0.39 ± 0.07	**0.038**
*Acinetobacter*	0.15 ± 0.06	0.09 ± 0.07	0.312

*P <* 0.05 are in bold

### Characterization of gut microbial community

**Fig. 3:**
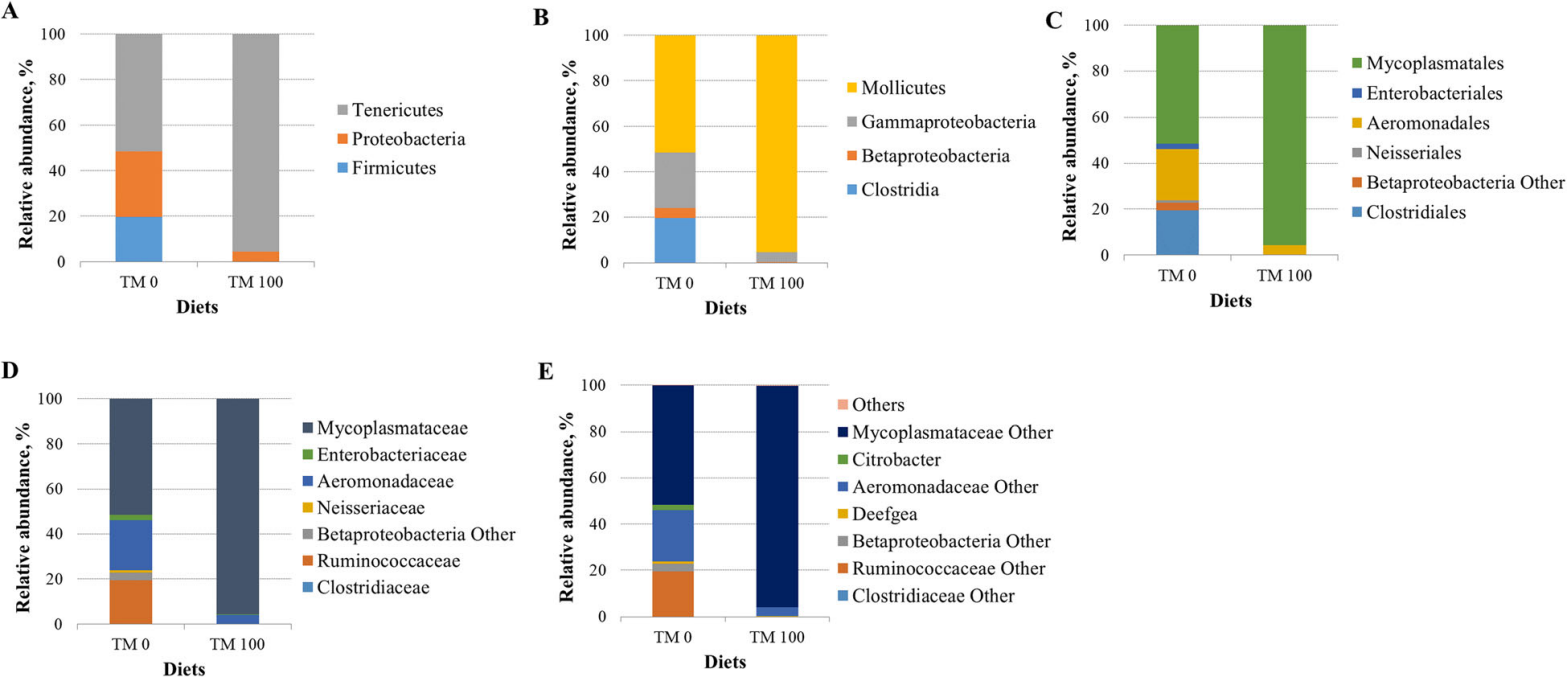
Relative abundance (%) of the overall most prevalent bacterial phyla (**a**), classes (**b**), orders (**c**), families (**d**), and genera (**e**) in GMMC. All bacteria with an overall abundance of ≥1% for **a**, **b** and **c;** and ≥ 0.5% for **d** and **e** were reported. Bacteria with lower abundance were pooled and indicated as “Others”

By taking into account all samples and considering only the most representative taxa, the gut microbial community of trout consisted of 3 phyla, 4 classes, 5 orders, 6 families, and 2 genera (Fig. 3; Table 5). Regardless of the diet, the most abundant phylum was Tenericutes*,* followed by Proteobacteria and Firmicutes in descending order of abundance. Among them, relative amount of Proteobacteria, mainly represented by Beta- and Gammaproteobacteria, was significantly influenced by diet (*P* = 0.047) resulting higher in control group (3-fold increase) (Fig. 3b, Table 5). At order level, trout fed with diet TM 100 showed a significantly four-fold decrease (*P* = 0.033) in Neisseriales, represented by Neisseriaceae family, compared to control trout (Fig. 3c; Table 5). The Ruminococcaceae family of Clostridiales order resulted detectable only in intestine of TM 0 fish (Fig. 3d; Table 5). No differences in relative abundances of intestinal bacterial genera were found in response to diet (Table 5).

**Table 5:** Mean of relative abundance (%) ± SD of the most prevalent phyla, classes, orders, families, and genera found in GMMC

Items	TM 0	TM 100	***P***-value
Phylum			
Firmicutes	19.51 ± 23.48	0.21 ± 0.19	0.747
Proteobacteria	29.00 ± 28.65	4.20 ± 5.20	**0.047**
Tenericutes	51.50 ± 38.26	95.56 ± 5.30	0.065
Class			
Clostridia	19.47 ± 23.43	0.18 ± 0.18	0.746
Betaproteobacteria	4.45 ± 5.15	0.05 ± 0.10	**0.012**
Gammaproteobacteria	24.55 ± 30.20	4.15 ± 5.12	0.336
Mollicutes	51.50 ± 38.26	95.56 ± 5.30	0.065
Order			
Clostridiales	19.47 ± 23.43	0.18 ± 0.18	0.746
Neisseriales	1.06 ± 1.12	0.05 ± 0.10	0.033
Aeromonadales	22.24 ± 30.74	3.98 ± 5.24	0.422
Enterobacteriales	2.27 ± 4.37	0.16 ± 0.37	0.144
Mycoplasmatales	51.50 ± 38.26	95.56 ± 5.30	0.065
Family			
Clostridiaceae	0.0 ± 0.0	0.2 ± 0.2	-
Ruminococcaceae	19.5 ± 23.4	0.0 ± 0.0	-
Neisseriaceae	1.1 ± 1.1	0.1 ± 0.1	**0.033**
Aeromonadaceae	22.2 ± 30.7	4.0 ± 5.2	0.422
Enterobacteriaceae	2.3 ± 4.4	0.2 ± 0.4	0.221
Mycoplasmataceae	51.5 ± 38.3	95.6 ± 5.3	0.065
Genus			
*Deefgea*	1.05±1.13	0.04±0.10	0.055
*Citrobacter*	2.20±4.38	0.00±0.00	-

*P <* 0.05 are in bold. “-” taxa detected only in one group

### Characterization of skin microbial community

**Fig. 4:**
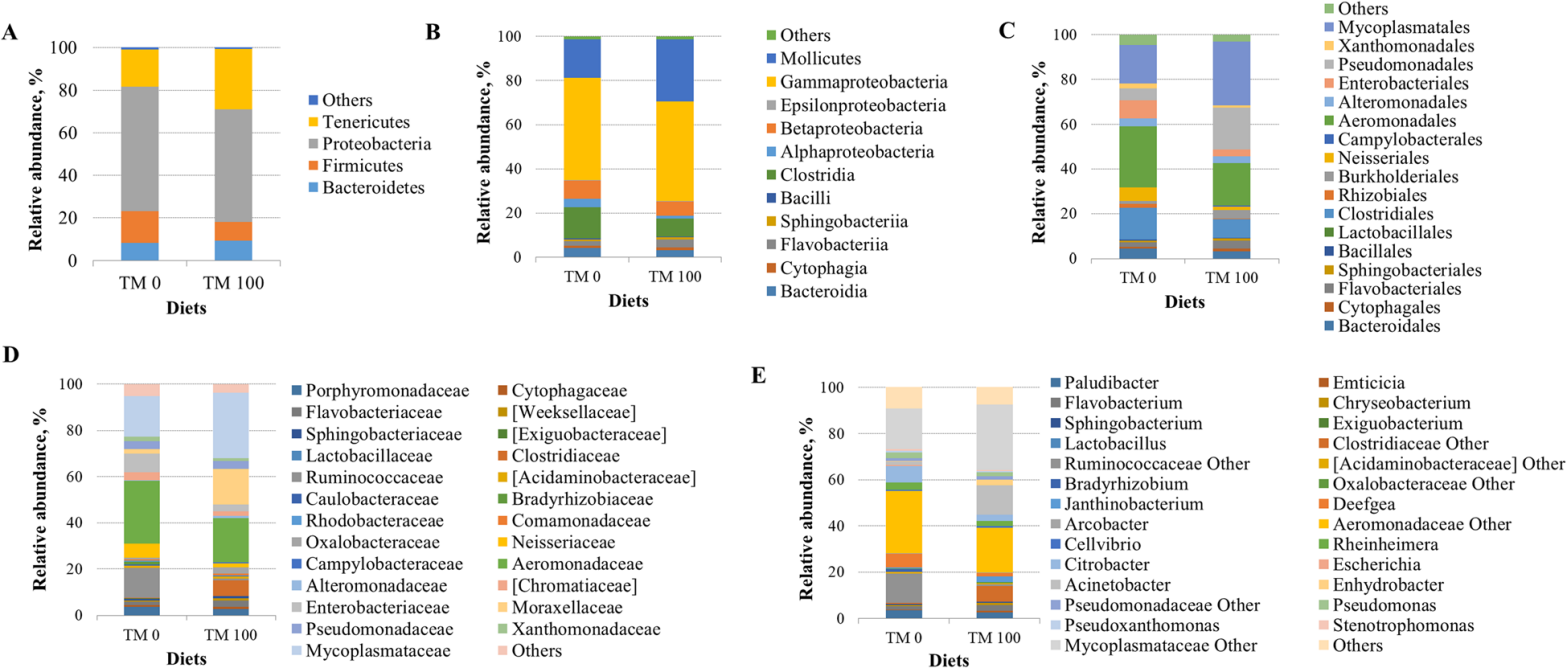
Relative abundance (%) of the overall most prevalent bacterial phyla (**a**), classes (**b**), orders (**c**), families (**d**), and genera (**e**) in GMMC. All bacteria with an overall abundance of ≥1% for **a**, **b** and **c;** and ≥ 0.5% for **d** and **e** were reported. Bacteria with lower abundance were pooled and indicated as “Others”

The skin microbial community was mainly consisted of 4 phyla, 11 classes, 17 orders, 25 families, and 20 genera (Fig. 4; Table 6). Regardless of the diet, the skin microbiome of trout was dominated by four phyla*:* Proteobacteria, Firmicutes, Tenericute*s*, and Bacteroidetes (Fig. 4a). At order level, the only difference between two groups was for Neisseriales, mainly represented by Neisseriaceae family, that were significantly higher (2-fold increase, *P*=0.013) in fish fed control diet (Fig. 4c; Table 6). At family level, Clostridiaceae resulted enriched (4-fold increase, *P*=0.013) in skin microbiota of trout fed with insect-based diet TM 100 (Fig. 4d; Table 6). Only genus Deefgea resulted significantly affected by diet (*P*=0.017), being two fold increased in control feeding group TM 0 (Fig. 4e; Table 6).

**Table 6:** Mean of relative abundance (%) ± SD of the most prevalent phyla, classes, orders, families, and genera found in SMMC

Items	TM 0	TM 100	***P***-value
Phylum			
Bacteroidetes	8.15 ± 6.71	9.28 ± 5.61	0.878
Firmicutes	14.82 ± 16.38	8.65 ± 7.18	0.810
Proteobacteria	58.77 ± 10.48	53.18 ± 20.76	0.617
Tenericutes	17.36 ± 9.49	28.19 ± 15.54	0.228
Class			
Bacteroidia	4.22 ± 4.88	3.17 ± 2.41	0.683
Cytophagia	0.80 ± 0.70	1.09 ± 0.82	0.802
Flavobacteriia	2.14 ± 1.21	3.74 ± 2.27	0.375
Sphingobacteriia	0.72 ± 0.49	0.93 ± 0.73	0.855
Bacilli	0.62 ± 0.63	0.29 ± 0.29	0.198
Clostridia	14.21 ± 16.56	8.35 ± 7.22	0.936
Alphaproteobacteria	3.80 ± 4.18	1.28 ± 0.90	0.153
Betaproteobacteria	8.17 ± 1.81	6.20 ± 4.24	0.247
Epsilonproteobacteria	0.18 ± 0.17	0.33 ± 0.25	0.397
Gammaproteobacteria	46.34 ± 7.74	45.12 ± 17.46	0.874
Mollicutes	17.36 ± 9.49	28.19 ± 15.54	0.228
Order			
Bacteroidales	4.22 ± 4.89	3.17 ± 2.41	0.683
Cytophagales	0.80 ± 0.70	1.09 ± 0.82	0.802
Flavobacteriales	2.14 ± 1.21	3.74 ± 2.27	0.375
Sphingobacteriales	0.72 ± 0.49	0.93 ± 0.73	0.855
Bacillales	0.39 ± 0.49	0.15 ± 0.18	0.211
Lactobacillales	0.23 ± 0.17	0.14 ± 0.11	0.275
Clostridiales	14.21 ± 16.56	8.35 ± 7.22	0.936
Rhizobiales	1.65 ± 3.49	0.30 ± 0.30	0.936
Burkholderiales	1.30 ± 1.51	3.60 ± 3.99	0.261
Neisseriales	5.94 ± 2.73	1.70 ± 2.45	**0.013**
Campylobacterales	0.18 ± 0.17	0.33 ± 0.25	0.397
Aeromonadales	27.21 ± 9.10	19.20 ± 9.11	0.154
Alteromonadales	3.69 ± 6.17	3.04 ± 2.49	0.969
Enterobacteriales	8.09 ± 5.16	2.96 ± 4.72	0.093
Pseudomonadales	5.43 ± 4.95	18.81 ± 23.79	0.471
Xanthomonadales	1.93 ± 1.68	1.12 ± 0.71	0.328
Mycoplasmatales	17.36 ± 9.49	28.20 ± 15.55	0.227
Family			
Porphyromonadaceae	3.64 ± 4.23	2.66 ± 2.17	0.636
Cytophagaceae	0.79 ± 0.71	1.08 ± 0.82	0.799
Flavobacteriaceae	1.50 ± 1.06	2.69 ± 1.87	0.368
[Weeksellaceae]	0.57 ± 0.29	0.99 ± 0.93	0.633
Sphingobacteriaceae	0.70 ± 0.48	0.90 ± 0.72	0.854
[Exiguobacteraceae]	0.13 ± 0.12	0.09 ± 0.11	0.367
Lactobacillaceae	0.02 ± 0.04	0.03 ± 0.06	0.549
Clostridiaceae	0.34 ± 0.21	6.68 ± 8.17	**0.013**
Ruminococcaceae	12.65 ± 17.33	0.63 ± 1.35	0.170
[Acidaminobacteraceae]	1.12 ± 1.48	0.95 ± 0.59	0.887
Caulobacteraceae	0.47 ± 0.53	0.30 ± 0.16	0.810
Bradyrhizobiaceae	1.15 ± 2.77	0.03 ± 0.03	0.683
Rhodobacteraceae	0.55 ± 0.62	0.16 ± 0.15	0.133
Comamonadaceae	0.48 ± 0.70	0.73 ± 0.42	0.433
Oxalobacteraceae	0.82 ± 0.81	2.87 ± 3.86	0.173
Neisseriaceae	5.94 ± 2.73	1.70 ± 2.45	**0.013**
Campylobacteraceae	0.18 ± 0.17	0.33 ± 0.25	0.397
Aeromonadaceae	27.21 ± 9.10	19.20 ± 9.11	0.154
Alteromonadaceae	0.31 ± 0.15	0.91 ± 1.55	0.936
[Chromatiaceae]	3.36 ± 6.09	2.11 ± 1.23	0.378
Enterobacteriaceae	8.09 ± 5.16	2.96 ± 4.72	0.092
Moraxellaceae	1.81 ± 1.60	15.47 ± 22.04	0.298
Pseudomonadaceae	3.62 ± 3.35	3.33 ± 2.08	0.818
Xanthomonadaceae	1.93 ± 1.68	1.12 ± 0.71	0.328
Mycoplasmataceae	17.36 ± 9.49	28.20 ± 15.55	0.228
Genus			
*Paludibacter*	3.60 ± 4.21	2.61 ± 2.15	0.629
*Emticicia*	0.21 ± 0.11	0.65 ± 0.53	0.197
*Flavobacterium*	1.49 ± 1.04	2.54 ± 1.92	0.440
*Chryseobacterium*	0.54 ± 0.31	0.92 ± 0.89	0.662
*Sphingobacterium*	0.44 ± 0.26	0.52 ± 0.47	0.964
*Exiguobacterium*	0.13 ± 0.12	0.09 ± 0.11	0.369
*Lactobacillus*	0.02 ± 0.04	0.02 ± 0.05	0.549
*Janthinobacterium*	0.54 ± 0.64	2.29 ± 3.78	0.378
*Deefgea*	5.85 ± 2.90	1.58 ± 2.48	**0.017**
*Arcobacter*	0.18 ± 0.17	0.33 ± 0.25	0.397
*Cellvibrio*	0.31 ± 0.15	0.91 ± 1.55	0.936
*Rheinheimera*	3.36 ± 6.09	2.11 ± 1.23	0.378
*Citrobacter*	7.11 ± 5.75	2.66 ± 4.54	0.471
*Escherichia*	0.53 ± 0.80	0.02 ± 0.05	0.253
*Acinetobacter*	1.46 ± 1.27	12.87 ± 18.51	0.185
*Enhydrobacter*	0.23 ± 0.24	2.32 ± 3.30	0.183
*Pseudomonas*	2.44 ± 2.13	1.92 ± 1.06	0.604
*Pseudoxanthomonas*	0.80 ± 1.13	0.41 ± 0.33	0.486
*Stenotrophomonas*	0.67 ± 0.50	0.43 ± 0.30	0.406

*P <* 0.05 are in bold

## Discussion

In the last decades, research on the use of insects as FM replacers in aquafeed is rapidly evolving. Several reviews have been published on insects nutritional value, environmental low impact, and food safety, all attributes that could contribute to make aquaculture system more productive and sustainable [6, 8, 9, 41].

In terms of fish growth, the research of our group, as also reported by Chemello et al. [36], confirms what has been found in previous studies, i.e. the complete or partial substitution of dietary fishmeal with TM does not affect rainbow trout growth performance and fillet quality [13–15]. Similarly, TM was successfully utilised and well accepted by several marine fish species [42–44]. While the effects of dietary FM/TM replacement on fish growth performances have been widely investigated, less evidence is available on the effects on host commensal bacterial communities. In particular, skin microbiome is underexplored in fish as well as in most farmed animals.

The data showed no major effects of FM substitution with TM meal on species richness and diversity of both gut mucosa- and skin mucus-associated bacteria. In line with our results, the inclusion of hydrolysed TM meal did not affect the total number of digesta-associated bacteria in sea trout (*Salmo trutta* m. *trutta*) [45]. In contrast, in the study of Józefiak et al. [46], the total number of intestinal bacteria increased in rainbow trout fed a diet in which FM was partially replaced by TM in comparison to control fish that were fed a FM-based diet.

Interestingly, Antonopoulou and colleagues [20] reported that the dietary inclusion of *T. molitor* larvae meal led to a five-fold increase of Simpson dominance D index, and to a two-fold decrease of the Shannon H index in rainbow trout gut microbiota, but not in sea bream and sea bass microbiota in which the same diversity indices remained practically unchanged. This evidence suggests a species-specific impact of insect meal on gut bacterial communities. Equally, in our previous studies, we found an increase of bacteria species richness and diversity in intestinal microbiome of trout fed diets with partial replacement of FM with *Hermetia illucens* meal [18, 19].

Regardless of the diet type, marked differences in terms of alpha diversity were found between gut and skin microbiota, being the latter characterized by higher microbial diversity and richness. Although these divergences could be partly due to the different rarefaction depth applied to compute alpha diversity, it is also true that previous studies on trout and other fresh water species displayed a similar trend with a lower alpha diversity in the gut than in the skin mucosal surface [27, 47, 48]. Unfortunately, in contrast to high number of studies focused on fish gut microbiome, the skin mucus microbiome remains largely underexplored.

Initially, fish skin is colonized by bacteria present in the water, but over time, the superficial mucus harbors an increasingly divergent microbial community [47, 49]. Like in intestine, the balance between members of skin microbial community, i.e., commensals, symbionts or pathogenic bacterial strains, collectively forming skin microbiome, is important to preserve fish health. It is well known that factors such as diet, water quality, seasonality, host physiology, infections, and stress can shape the composition of fish microbiomes and influence the balance of the microbic ecosystems [33–35].

Our metabarcoding analysis showed that rainbow trout skin microbiome was largely dominated by Proteobacteria, and especially Gammaproteobacteria, which constituted approximately half of the bacterial taxa found. This result is in agreement with previous studies on other fish species regardless of the technique used for bacterial identification [26–28, 30, 31, 50–52]. Gammaproteobacteria class includes several potentially pathogenic bacterial species for fish, such as *Vibrio anguillarum*, and *Photobacterium damselae*. Actually, there are several evidences supporting the role of fish skin microbiota as an important niche for mucosal pathogen evolution in nature [50]. For instance, potentially pathogenic *Vibrio*, such as *Vibrio anguillarum* and *Vibrio cholerae*, monopolize skin microbiome of wild eel (*Anguilla anguilla*) from estuary and wetland [50]. Other accidental pathogens identified in wild eel have been *Pseudomonas aeruginosa*, *Stenotrophomonas maltophilia*, *Achromobacter xylosoxidans*, and *Aeromonas veronii*. Similarly, skin microbiome of coral reef fish showed a significant enrichment in Gammaproteobacteria, especially Vibrionaceae [31].

Although in the present study trout skin microbiome was dominated by the Gammaproteobacteria’s family of Aeromonadaceae instead of Vibrionaceae, at genus level, *Pseudomonas*, *Stenotrophomonas* and *Citrobacter* were present in our samples likewise in wild and farmed eel skin microbiome [50]. This result is quite interesting, since previous studies have indicated that fish skin microbiome is species-specific, both in terms of bacterial diversity and bacterial community structure, showing significantly lower variability between individuals from the same species than between those of different species [26, 31].

The low frequency of *Vibrio* genera in trout skin microbial community could be explained by the fact that trout is a freshwater fish while *Vibrio* are mainly marine bacterial genera. It is widely accepted, indeed, that the skin of fish harbors a complex and diverse microbiota that closely interacts with the microbial communities of the surrounding water.

In line with our data, Lowrey et al. [27] reported that Proteobacteria and Bacteroidetes were the most abundant phyla of rainbow trout skin microbiota, however at genus level they found a skin bacterial community consistently composed by *Flectobacillus*. These apparently controversial evidences are inevitable since, up to date, few studies have investigated skin microbiome in freshwater fish, and it is not yet known if it fundamentally differs from that of marine fish [51].

With regard to skin microbial community composition, the two dietary groups did not display distinctive features, except for a decrease in the relative abundance of *Deefgea* genus (family Neisseriaceae) in skin microbiome of trout fed with insect meal. Changes in the skin microbiota of fish in response to stressors, such as hypoxia have been previously observed, in brook charr (*Salvelinus fontinalis*), in which probiotic-like bacteria decreased after stress exposure [53]. Studies in salmonids have also shown that parasitic infections or other microbial aetiological agents (e.g. viruses) may perturb skin microbiota [30].

In agreement with our recent study in rainbow trout [19], metagenomic analysis indicated that Tenericutes was the most abundant phylum in trout intestine, regardless of the diet. Specifically, within this phylum, the Mollicutes, mainly represented by Mycoplasmataceae family, were the dominant class. The Tenericutes are among the protagonists of gut symbionts of rainbow trout, indicating that they are possibly related to the metabolism of the host [27, 54, 55]. Although diet is the most important external factor affecting the gut microbiota composition, in this case we observed only a weak dietary modulation of intestinal bacterial communities. The only changes due to dietary FM substitution with TM meal were a decreased number of Proteobacteria and, at family level, a reduced number of taxa assigned to Ruminococcaceae and Neisseriaceae.

In line with our results, Antonopoulou et al. [20] reported that *T. molitor* meal replacement affected the dominant intestinal phyla less in rainbow trout than in sea bream and sea bass. In contrast, there are several evidences that FM replacement with insect meal from black soldier fly (*Hermetia illucens*) larvae positively modulates gut microbiota of rainbow trout by increasing the proportion of lactic acid bacteria (LAB), which are generally considered as beneficial microorganisms and frequently used as probiotics in fish and other vertebrates diet [18, 19, 56].

Actually, there is a study stating that the inclusion of 20% TM meal in the diet increased the intestinal population of *Lactobacillus* and *Enterococcus* genera in rainbow trout juveniles [23]. The increase of LAB by dietary insect meal could be related to the prebiotic properties of chitin. Chitin is an insoluble linear polysaccharide (a biopolymer of N-acetyl-β-*D*-glucosamine) that confers structural rigidity to insects’ exoskeleton. Partial or full enzymatic deacetylation of chitin produces chitosan. Both chitin and chitosan are hardly digested by the majority of fish [21]; therefore, once consumed, the fermentation of both polysaccharides is largely performed by gut microbiota. The lack of enrichment in intestinal LAB during the present study was an unexpected result, especially when compared to what has been previously observed in the intestine of trout fed with diets containing *H. illucens* larvae meal [18, 19]. The main effect of the dietary inclusion of this type of insect meal was a significant increase of Firmicutes at the expense of Proteobacteria phylum. The dietary administration of TM meal caused instead only a decrease in relative amount of Proteobacteria without any increase in Firmicutes.

## Conclusions

In summary, the data demonstrated that yellow mealworm (*T. molitor*) larvae meal is a valid alternative animal protein to replace FM in the aquafeeds. In summary, the data demonstrated that yellow mealworm (*T. molitor*) larvae meal is a valid alternative animal protein to replace FM in the aquafeeds. The totally replacement of FM with TM did not cause negative effects on rainbow trout gut and skin microbial communities. No evident sign of dysbiosis was detected, but only slight microbiota changes after total FM substitution with insect meal. Specifically we assisted to a reduction in relative abundance of Neisseriaceae bacterial family, in both gut and skin. Differences at genus level were identified only at the skin leveln with a two-fold decrease of Deefgea genus in trout fed with TM 100 diet. Last, but not least, the mapping of the trout skin microbiota represents a novel contribution of the present study since fish skin microbiota is still scarcely investigated, in particular in freshwater fish. Indeed, in contrast to the increasing knowledge on gut microbiota, the skin microbiota of major farmed fish species remains largely unmapped but it deserves thorough consideration.

## Data Availability

All DNA sequencing data were deposited as FASTQ files at the European Nucleotide Archive (EBI ENA) public database under the accession code: **PRJEB38845.**

## Abbreviations

FM: Fishmeal
TM: *Tenebrio molitor*
GMMC: Gut mucosa microbial communities
SMMC: Skin mucosa microbial communities
LAB: Lactic acid bacteria
